# Induction of a specific CD8+ T-cell response to cancer/testis antigens by demethylating pre-treatment against osteosarcoma

**DOI:** 10.18632/oncotarget.2505

**Published:** 2014-10-04

**Authors:** Binghao Li, Xiaobing Zhu, Lingling Sun, Li Yuan, Jian Zhang, Hengyuan Li, Zhaoming Ye

**Affiliations:** ^1^ Department of Orthopaedics, The Second Affiliated Hospital of Zhejiang University School of Medicine, Hangzhou, 310008, China; ^2^ Department of Orthopaedics, Taizhou Cancer Hospital, Taizhou, 317502, China; ^3^ School of Public Health, Fudan University, Shanghai, 200032, China; ^4^ Centre for Orthopaedic Research, Department of Orthopaedics, The Second Affiliated Hospital of Zhejiang University School of Medicine, Hangzhou, 310008, China

**Keywords:** Osteosarcoma, Adoptive immunotherapy, Cancer/testis antigens, Demethylating treatment, Combination therapy

## Abstract

Conventional non-surgical therapeutic regimens against osteosarcoma are subject to chemoresistance and tumor relapse, and immunotherapy may be promising for this tumor. However, it's hard to find satisfactory epitopes for immunotherapy against osteosarcoma. Cancer/testis antigens (CTAs), such as MAGE-A family and NY-ESO-1, the potential antigens that almost exclusively express in tumor cells and immune-privileged sites, have been found expressed in osteosarcoma also. Nevertheless, the expression of CTAs is downregulated in many tumors, constraining the application of immunotherapy. In this article, we demonstrate that the expression of MAGE-A family and NY-ESO-1 in osteosarcoma cells can be upregulated following treatment with demethylating agent 5-aza-2′-deoxycytidine and consequently induces a CTA specific CD8+ T-cell response against osteosarcoma *in vitro* and *in vivo*. The *in vivo* imaging was realized by using luciferase-transfected HOS cells and DiR labeled T-cells in severely combined immunodeficiency mouse models. Cytotoxic T cells specifically recognizing MAGE-A family and NY-ESO-1 clustered at the tumor site in mice pre-treated with DAC and resulted in tumor growth suppression, while it was not observed in mice without DAC pre-treatment. This study is important for more targeted therapeutic approaches and suggests that adoptive immunotherapy, combined with demethylating treatment, has the potential for non-surgical therapeutic strategy against osteosarcoma.

## INTRODUCTION

Osteosarcoma (OS) is the most common malignant tumor of bone in childhood and adolescence, which is also associated with local invasion and early metastatic potential [1]. Despite intensification of conventional chemotherapy and surgery, the 5-year survival rate for patients with localized tumor have reached a plateau at about 65% [2], and it is 20% for patients with relapsed tumors or metastasis [3]. There has been very little improvement in the outcomes of patients with localized osteosracoma since 1980s. Therefore, alternate therapeutic strategies are needed not only for patients with refractory tumors but also as an adjuvant to localized diseases.

Immunotherapy has been considered to be a promising method against osteosarcoma [4]. The immune system plays an important role in controlling osteosarcoma. Transfer of naive T cells to murine osteosarcoma models resulted in immune reconstitution and significantly decreased metastatic recurrence [5]. Therefore, immunotherapy based on upregulation of immune response will be an encouraging therapeutic strategy against osteosarcoma [6]. In consideration of the significant toxic side effects caused by the use of cytokines in active immunotherapy, adoptive immunotherapy targeting tumor specific antigens by CD8+ T lymphocytes may be promising. Finding a tumor specific antigen that can be reasonably targeted by CD8+ T cells is a key component in the application of adoptive immunotherapy for osteosarcoma. Cancer/testis antigens (CTAs), such as MAGE-A family proteins and NY-ESO-1, are considered to exist only in tumor cells including osteosarcoma [7], glioblastoma [8], breast cancer [9], and immune-privileged sites [10]. There are also meta-analysis studies for CTAs as biomarkers in tumors [11, 12]. The specificity of CTAs makes them brilliant epitopes for antigen specific CD8+ T lymphocytes. Nevertheless, the level of CTA expression may vary widely in tumors, and some CTA genes are silenced in osteosarcoma, complicating the CTA-based immunotherapy. If CTA expression may be elevated in osteosarcoma, the increased tumor immunogenicity will create excellent conditions for CTA specific immunotherapy.

Epigenetic mechanisms play important roles in regulating CTA expression, especially DNA methylation [13]. Methylation on promoter will result in the downregulated expression of genes; demethylating agents, such as decitabine (5-aza-2′-deoxycytidine, DAC), are potent inhibitors of DNA methylation. A substantial number of studies proved that demethylating treatment would lead to reexpression or enhanced expression of CTAs in multiple tumors, including leukemia [14, 15], pancreatic cancer [16], and neuroblastoma [17], *etc.* More data on osteosarcoma are still urgently needed.

In this study, we have demonstrated that expression of MAGE-A family and NY-ESO-1 in osteosarcoma cell line U2OS and HOS can be elevated following demethylating treatment with DAC. Among them, NY-ESO-1, MAGE-A10 and MAGE-A4 are the most upregulated in U2OS; NY-ESO-1 and MAGE-A4 are the most upregulated in HOS. Furthermore, we generated CTA specific T-cells *in vitro* and proved that the elevated CTA expression would facilitate CTA specific CD8+ T-cell-mediated tumor cell killing *in vitro* and *in vivo*.

## RESULTS

### Demethylating treatment showed time-dependent effects on elevating CTA expression in U2OS and HOS over a 7-day interval

**Figure 1:**
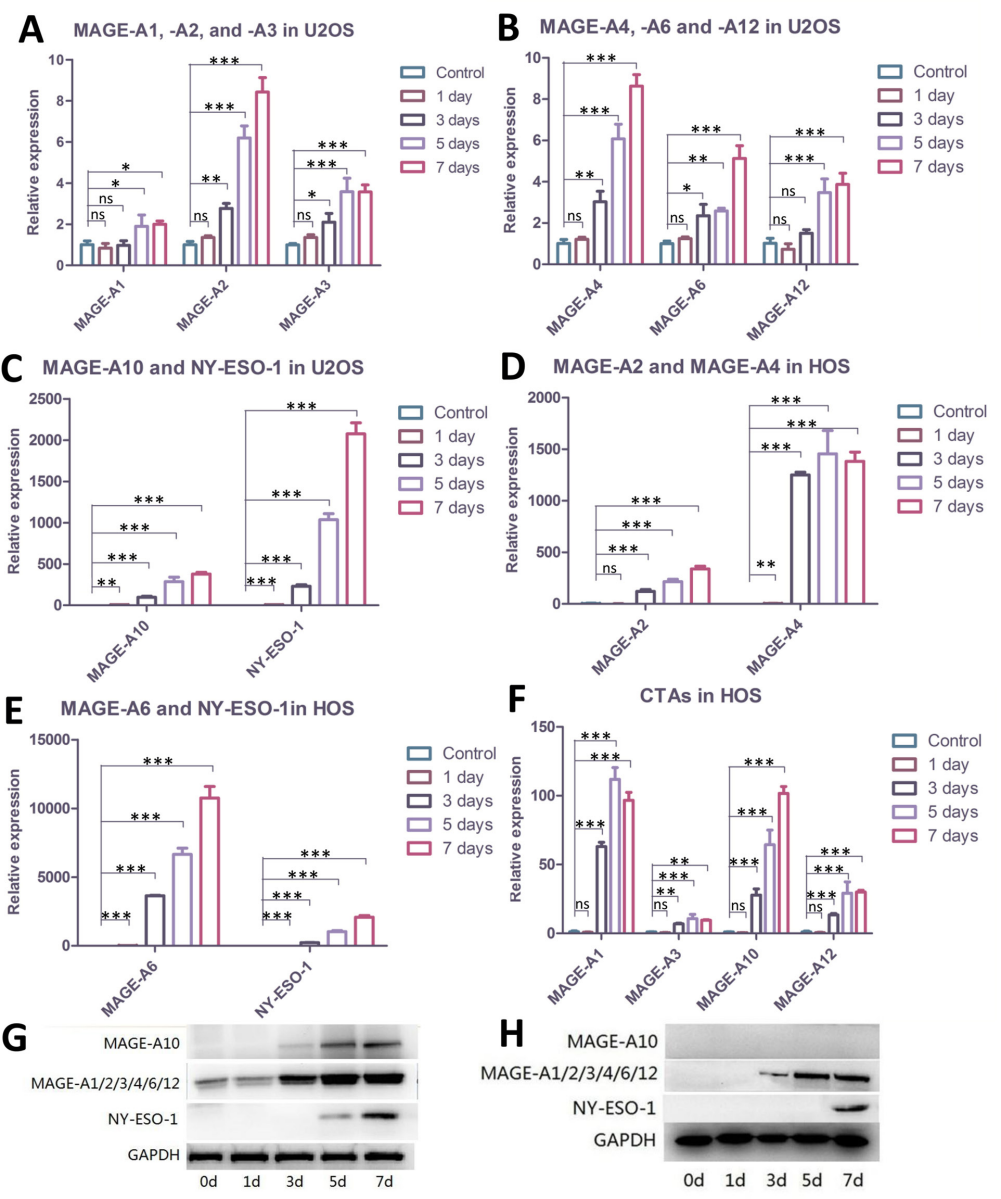
Demethylating treatment elevated the expression of CTAs in U2OS and HOS cells **(A, B, C)** qRT-PCR showing levels of CTAs in U2OS cells before or after demethylating treatment. Among the eight assessed CTAs, NY-ESO-1 and MAGE-A10 represented the largest magnification; the rest of the antigens also showed elevation. **(D, E, F)** qRT-PCR demonstrating expression of CTAs in HOS cells was elevated post-treatment. MAGE-A4, -A6 and NY-ESO-1 showed the largest promotion in expression; the rest five antigens also represented enhanced expression in different degrees after demethylating treatment. **(G)** Western blots on U2OS cells. MAGE-A10 and NY-ESO-1 were expressed after 5 days of demethylating treatment. At least two of the MAGE-A proteins were already expressed in untreated cells; elevation in expression, however, was also observed. **(H)** Western blots on HOS cells. MAGE-A proteins started to express after three days of treatment while NY-ESO-1 took seven days. MAGE-A10 protein was undetectable throughout the treatment, although elevation at mRNA level was observed. **Error bars** represent Standard Deviation; * *p* < 0.05, ** *p* < 0.01, *** *p* < 0.001.

We treated the cells with 1 μM DAC for 1 day, 3 days, 5 days and 7days respectively, and then cells were harvested to perform western blot and quantitative real-time PCR (qRT-PCR). Data presented in Figure 1a, 1b, 1c and 1g demonstrated that DAC showed time-dependent effects in elevating CTA expression in U2OS cells, albeit some of the CTAs tested in this study are already expressed in this cell line. Among them, the expression of MAGE-A10 and NY-ESO-1 showed the strongest enhancement in both protein and mRNA level. MAGE-A10 and NY-ESO-1 proteins were undetectable in untreated U2OS cells, and the expression of mRNA was also at a low-level. As treatment time increased, however, the expression of each of these CTAs was significantly enhanced. In untreated cells, MAGE-A1 and MAGE-A4 showed the strongest expression at mRNA level. As expected, the result of western blot assay showed that MAGE-A proteins were already expressed in U2OS cells, although it was difficult to identify exactly which ones were detected. According to a previous study, the immunoblot analysis revealed that MAGE-A1 and MAGE-A4 proteins were expressed in U2OS cells [7], which were consistent with our results of qRT-PCR for these two roles. Nevertheless, demethylating treatment strengthened the expression of these two antigens, especially MAGE-A4. The expression of MAGE-A2, A3, A6 and A12 were also enhanced in different degrees, which may elevate the immunogenicity of U2OS cells to T-cells.

Although expression of CTAs is already found in U2OS cells, none of the CTAs we examined in this study are expressed in untreated HOS cells. Encouragingly, successful enhancement of CTA expression was induced after demethylating treatment. Data presented in Figure 1d and 1e demonstrated that expression of MAGE-A4 and NY-ESO-1 was significantly elevated. There was expression of MAGE-A4 after 3 days of exposure to DAC, and NY-ESO-1 was expressed after 7 days of treatment (figure 1h). The expression of MAGE-A6 seemed to represent a dramatic magnification compared to the control group; however, it was still weaker than that of MAGE-A4 or NY-ESO-1. This may be due to the extremely low expression level of MAGE-A6 in untreated cells. We repeated the assays several times and checked the expression of MAGE-A6 in original HOS cells, and finally confirmed it. The rest of CTAs also showed an elevated expression in different degrees.

### CTA specific CD8+ T-cells were generated, and recognized DAC-treated osteosarcoma cells *in vitro*

**Figure 2:**
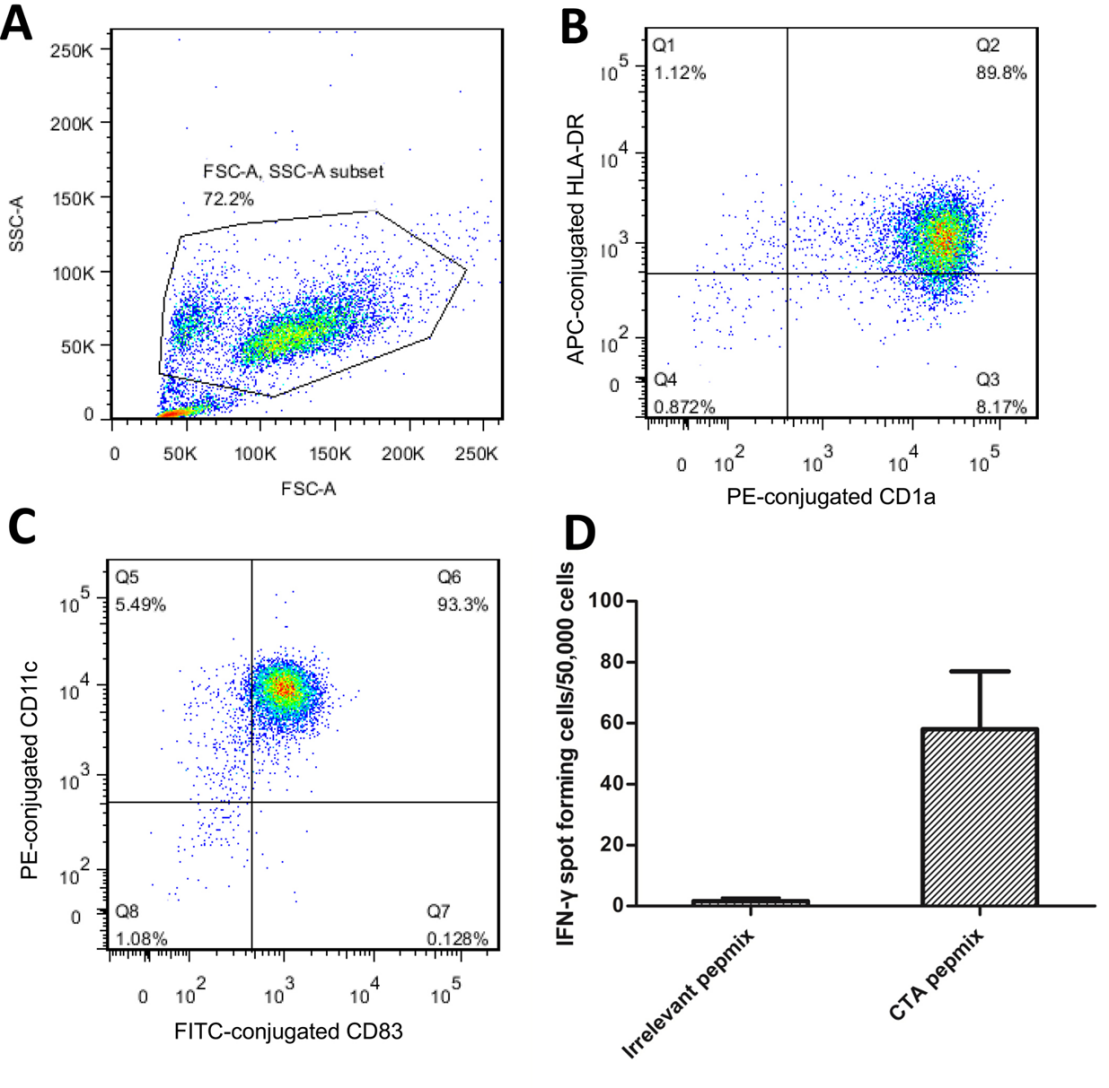
Identification of the generated DCs and CTA specific T-cells **(A)** Gate on tested cells. **(B, C)** Phenotypic analysis of the mature DCs. Typical expression of mature DC: CD1a+, CD11c+, CD83+ and HLA-DR+. At least 89.8% **(B)** of the gated cells were mature DCs. **(D)** Specificity of the generated CTA specific T-cells was assessed by IFN-γ ELISpot. A median response of 58 IFN-γ spot forming cells/50000 cells (range: 40–82) was observed in response to CTA peptides, compared with a median of 2 spot forming cells/50000 cells (range: 0–3) in response to irrelevant peptides. **Error bars** represent Standard Deviation.

**Figure 3:**
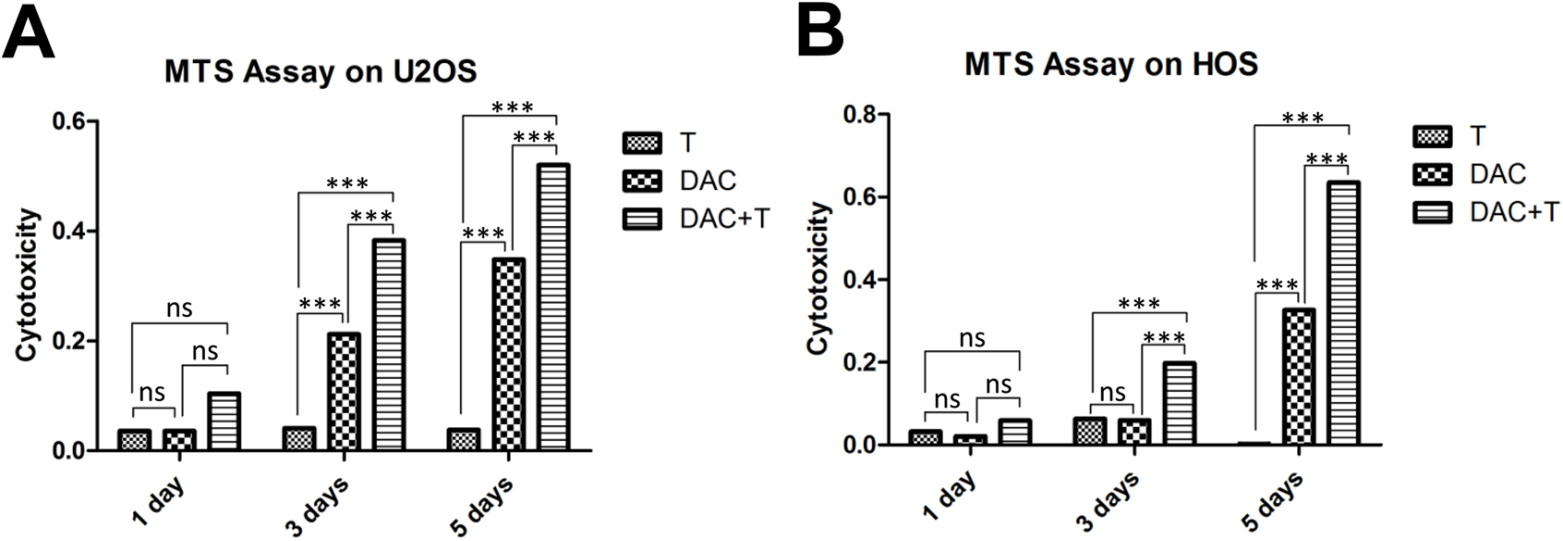
Cytotoxic assay using MTS assays to evaluate the cytotoxic effects of the generated CTA specific T-cells against osteosarcoma cells **(A)** MTS assay on U2OS cells. Throughout the treatment, treatment with T-cells alone represented no effect on tumor cell suppression. DAC treatment alone inhibited the growth of U2OS cells; however, T-cells showed more efficiency in combination with DAC pre-treatment, albeit the failure in monotherapy. **(B)** MTS assay on HOS cells. Similar to the results of U2OS cells, immunotherapy mediated by CTA specific T-cells showed synergistic effect with demethylating treatment. *** p < 0.001, ns refers to not significant in statistical differences.

In consideration of the existing expression of MAGE-A antigens in U2OS cells, DCs utilized in the assays against U2OS cells were pulsed with NY-ESO-1 peptide only. In order to culture CD8+ T-cells, which would recognize each MAGE-A antigen individually for the use in cytotoxic assays against HOS cells, we synthesized peptide p248V9 (YLEYRQVPV) [18]. Using previously generated DCs pulsed with multi-MAGE-A and NY-ESO-1 peptide mix, broad-spectrum-recognizing CD8+ T-cells were stimulated from PBMCs, and subsequently expanded rapidly with IL-2. Phenotype analysis of the generated DCs by flow cytometry was illustrated in figure 2a, 2b, and 2c; and the specificity of stimulated T-cells was assessed by ELIspot (figure 2d). A median response of 58 IFN-γ spot forming cells/50000 cells (range: 40–82) was observed in response to CTA peptides, compared with a median of 2 spot forming cells/50000 cells (range: 0–3) in response to irrelevant peptides. In MTS assays, the CTA specific CD8+ T-cells showed cytotoxicity against U2OS and HOS cells pre-treated with DAC (figure 3), while there was hardly any effect on untreated tumor cells. DAC treatment showed time-dependent cytotoxic effects, consistent with the results of a previous study on the single agent effects of DAC on osteosarcoma [19].

### Demethylating treatment was able to induce CTA specific CD8+ T-cell-mediated killing against osteosarcoma *in vivo*

**Figure 4:**
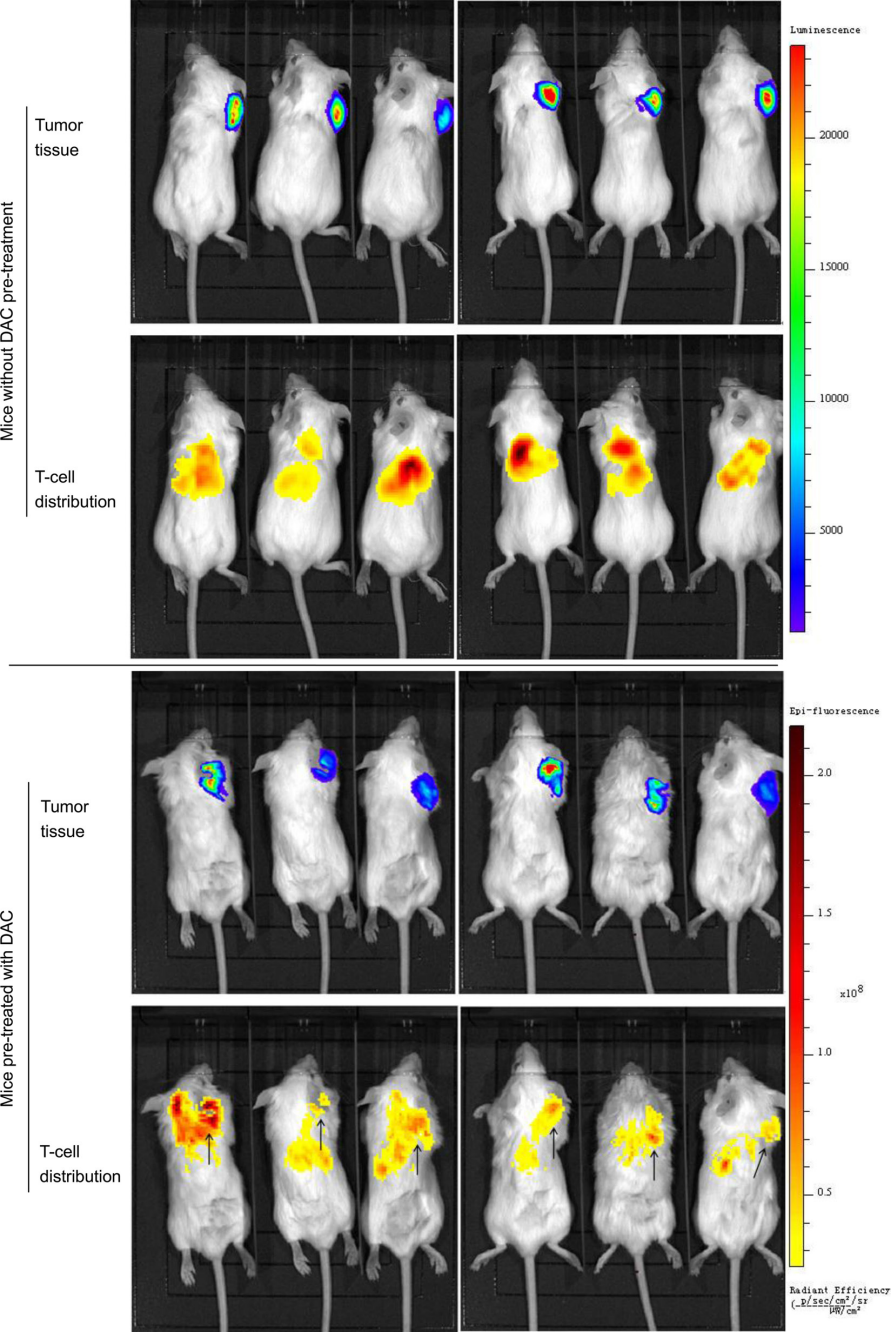
*In vivo* imaging of antitumor activity of CTA specific T-cells in xenograft models HOS cells transfected with luciferase (HOS-Luc) and SCID mice were used to establish animal models. Mice were imaged 24 hours postinjection of T-cells. Bioluminescence by HOS-Luc cells and fluorescence by DiR labeled T-cells were visualized with imaging system. In mice without DAC pre-treatment, T-cells distributed in liver, lung and scar of injection sites, while no signal was detected around the tumor tissue. In mice treated with DAC, T-cells clustered at the tumor site in addition to liver and lung. Demethylating treatment promoted the response of CTA specific T-cells to osteosarcoma tissue.

**Figure 5:**
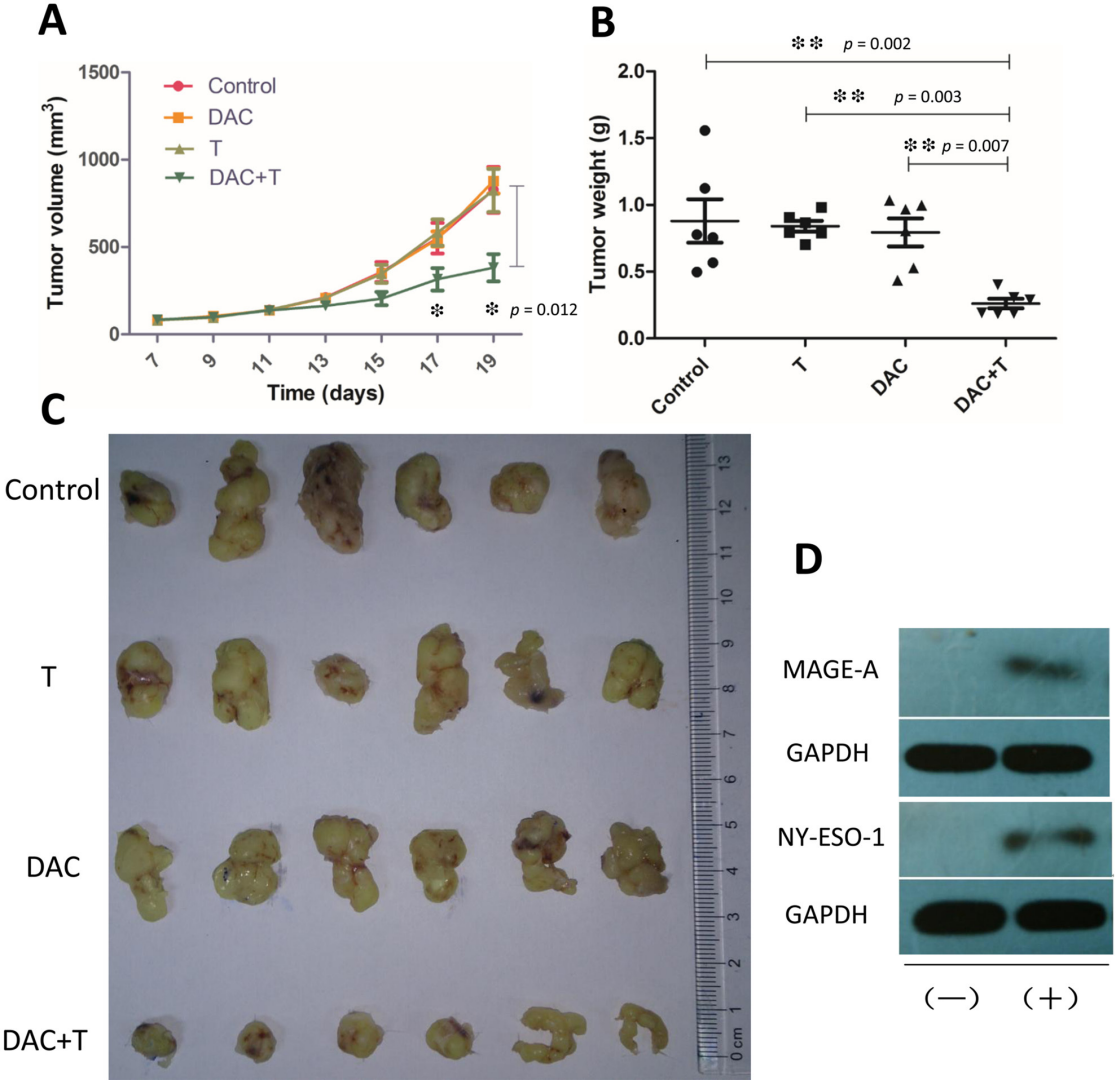
Effects of *in vivo* treatment on osteosarcoma **(A)** Tumors were measured with caliper every two days, starting on the 7^th^ day. Treatment with DAC was administrated from the 7^th^ day to the 11^th^ day, and mice started to receive *i.v.* injection of CTA specific T-cells every two days from the 12^th^ day. In mice pre-treated with DAC, T-cells represented high efficiency in inhibiting tumor growth. Statistical difference in tumor volume was shown the 17^th^ day, while there was no statistical difference among the other 3 groups. **(B)** Weights of the xenografts from **(C)** on the 19^th^ day. **(C)** Xenografts excised from the tumor bearing mice on day 19. **(D)** Western blot showed that expression of MAGE-A and NY-ESO-1 was elevated after *in vivo* treatment with DAC. (+) : Treated with DAC; (−) : Without DAC treatment. **Error bars** in **(A)** and **(B)** refer to Standard Error of Mean.

Tumor tissues in DAC treated SCID mice showed CTA expression, which was demonstrated in western blotting (figure 5d), while CTA was not detectable in untreated xenografts. Albeit the expression of CTAs was so weak that we failed in the illustration by immunohistochemistry (not shown in this paper), we observed the target band on the film by long exposure. The weak but indeed existing expression of CTAs successfully induced antigen specific CD8+ T-cell response to the tumor. Utilizing the *in vivo* imaging system, we detected the bioluminescence by luciferase-transfected tumor tissue and the fluorescence by DiR labeled T cells. In DAC pre-treated mice, the injected T-cells clustered at the tumor site in 24 hours, while in mice without DAC pre-treatment, they were only found in the lung and the liver (figure 4). The T-cells also inhibited tumor growth in DAC pre-treated mice, while demethylating treatment or immunotherapy alone showed no anti-cancer effect on mice in the two groups left (figure 5a and 5b). On the 19^th^ day, the mean volume of tumor xenografts in mice treated with T-cells in combination with DAC was 388.1 mm^3^ (range: 172 mm^3^–700 mm^3^), while it was 827.7 mm^3^ in mice without any treatment (range: 416 mm^3^–1250 mm^3^), 878.3 mm^3^ in mice with DAC treatment alone (range: 650 mm^3^–1090 mm^3^) and 824 mm^3^ in mice took immunotherapy alone (range: 405 mm^3^–1250 mm^3^). The mean weight of tumor xenografts in mice treated with both therapies was 0.261g (range: 0.190g–0.403g), and it was 0.880g in control group (range: 0.498g–1.558g), 0.841g in mice with DAC treatment alone (range: 0.705g–0.982g) and 0.795g with T-cells alone (range: 0.435g–1.035g). These results indicate that CTA specific adoptive immunotherapy in combination with demethylating treatment have anti-tumor effects on osteosarcoma.

## DISCUSSION

Osteosarcoma is a particularly aggressive cancer and attempts in changing chemotherapy regimens for poor responders have generally failed. Immunotherapy is considered to be a promising method in treatment against osteosarcoma, especially in adjuvant therapy. Several groups performed immunotherapy against osteosarcoma using NK cells and reported that osteosarcoma cells were potentially susceptible to NK cell cytotoxicity [20, 21]; studies focusing on using NK cells against osteosarcoma are going on [22]. Specific immunotherapy targeting HER2 with modified T cells against osteosarcoma was also conjectured to be feasible, and a substantial number of attempts were performed to resolve the issue of osteosarcoma cells expressing HER2 at a too low level [23, 24]. In addition, in our previous study, we demonstrated that osteosarcoma cells were highly susceptible to γδ T cell-mediated killing following treatment with zoledronate [25], which is a widely used amino bisphosphonate in inhibiting osteoclastic bone resorption in advanced cancers [26] and shows anti-tumor effects on osteosarcoma by inducing apoptosis and inhibiting proliferation [27, 28]. Nevertheless, it was still restricted to immune escape as the shortcoming in recognition. Pharmacologic upregulation of specific antigens may be a promising method to maximize the response to specific immunotherapy.

Cancer/testis antigens are characterized by specificity in tumors, several clinical trials using NY-ESO-1 or MAGE-A3 specific lymphocytes against soft-tissue tumors and lung cancer have achieved success [29–31]. However, some CTAs are epigenetically downregulated in tumors, complicating the administration of targeted immunotherapy. There has been abundant evidence that exposure to demethylating agents can lead to expression of CTAs in different tumor cell lines. Of note, this effect on CTA genes appears to be selective, with preferential upregulation in tumors [32], which may minimize the risk of autoimmunity. In recent years, use of overlapping peptide library spanning the MAGE-A proteins or NY-ESO-1 may successfully expand CTA specific T-cells from healthy donors or patients *in vitro* [33, 34]. In consideration of the fact that individual MAGE-A expression varies from one tumor to the other, a high-affinity heteroclitic peptide p248V9 that derived from MAGE-A1, A2, A3, A4, A6, A10, A12 was found [35]. This peptide may stimulate T cells that are able to recognize each of the seven MAGE-A proteins, which will facilitate the application of MAGE-A based adoptive immunotherapy. As long as osteosarcoma cells express at least one of the CTAs listed above, specific T-cells may be stimulated by the peptide library and consequently apply immunotherapy.

DAC was proved to induce apoptosis in osteosarcoma, and the dose used in that study was 1 μM [19], which was the reason why we selected this dose in our *in vitro* study. DAC will induce apoptosis through epigenetic mechanisms, which are independent of known mechanisms that conventional cytotoxic drugs take effect in osteosarcoma. Although the single agent effect of DAC on solid tumors appeared to be less than desirable in several clinical trials [36–38], it may have a place in adjuvant therapy or combination therapy [36]. DAC was already approved by the U.S. FDA in 2006 for use in the treatment of several leukemias, and sufficient assessments of drug safety have been made already. Successful combination treatment of demethylating therapy and adoptive immunotherapy against leukemias also attracted attention. A successful induction of CD8+ T-cell response to MAGE by demethylating treatment against acute myeloid leukemia and myelodysplasia was shown in 2010 [14] and, subsequently, DAC was demonstrated to successfully enhance the MAGE-A4 specific T-cell immune response in patients with relapsed Hodgkin lymphoma in 2011 [15]. Moreover, DAC was considered to have minimal effects on T-cell phenotype and function [15], and its effect on elevating the expression of CTAs appeared to be selective [16, 39]. Therefore, DAC may be utilized to increase tumor immunogenicity in CTA specific immunotherapy.

In this study, we generated broad-spectrum CTA specific T-cells with the peptide mix and employed them in combination with demethylating agent DAC for cancer treatment. Demethylating treatment enhanced the expression of CTAs *in vitro* and *in vivo*, while other roles related with CD8+ T-cell-mediated specific immune response, such as major histocompatibility complex molecules-I (MHC-I) and intercellular cell adhesion molecule-1 (ICAM-1), were found not changed after demethylating treatment in our study (not shown in this paper). In studies on mice, treatment with DAC alone didn't show any obvious effect on inhibiting tumor growth. This may be correlated with the low dose of DAC (1μg/g of body weight) that we used in demethylting treatment, while it was 2.5μg/g of body weight in the previous study [19]. Demethylating treatment facilitated the recognition of T-cells to tumor, and consequently inhibited tumor growth. Therefore, we hold the view that the elevated expression of CTAs may be one of the most important elements in enhancing CTA specific immunotherapy against osteosarcoma. It is particularly worth mentioning that although no distinct side effect was observed in our *in vivo* study, a previous research attracted focus on the potential risk in using broad-spectrum recognizing T-cells with high-avidity T-cell receptors (TCRs) in clinical trials [40]. MAGE-A12 was revealed to be expressed in rare neurons, *i.e.* there may be neurological toxicity in some individuals following treatment with T-cells recognizing epitopes in MAGE-A12 [40]. Albeit trials using T-cells recognizing NY-ESO-1 or the other MAGE-A proteins reported good news and no evidence was found that the other CTAs might be expressed in normal tissues except for immune-privileged sites, we have to be cautious enough while using broad-spectrum recognizing T-cells in clinical trials. Taking the previous clinical trials and our results into consideration, we hold the view that NY-ESO-1 and MAGE-A4 could possibly be safe and feasible targets for T-cells in further clinical trials on osteosarcoma.

In conclusion, the results presented here indicate that demethylating treatment with DAC against osteosarcoma cells can lead to an obvious enhancement in expression of CTAs, which may be brilliant targets for adoptive immunotherapy; while MHC-I and ICAM-1 were not changed. NY-ESO-1 and MAGE-A4 expression was significantly upregulated in both cell lines, and MAGE-A10 expression was also enhanced in U2OS. The efficacy of DAC-mediated enhancement of CTA specific immune response was determined by cytotoxic assays and *in vivo* studies. Our results highlight the synergistic role that demethylating treatment could play with specific immunotherapy in the control of osteosarcoma. Targeted immune-based treatment could be considered to be a promising strategy for patients with relapsed or refractory osteosarcoma.

## MATERIALS AND METHODS

### Ethics statement

Investigation has been conducted in accordance with the ethical standards and according to the Declaration of Helsinki and according to national and international guidelines and has been approved by the authors' institutional review board.

### Cell cultures and treatment

The human osteosarcoma cell lines U2OS (CTAs are expressed) and HOS (CTAs are undetectable) were purchased from the Cell Collection of Chinese Academy of Science (Shanghai, China). U2OS cells were cultured in RPMI 1640 medium (Gibco, Rockville, USA), whereas HOS cells were maintained in Dulbecco's modified Eagle's medium (DMEM) (Gibco). All media were supplemented with 10% fetal bovine serum (Invitrogen, Carlsbad, USA) and 100μg/ml streptomycin-penicillin. All cells were incubated at 37°C in 5% CO_2_. Cells were counted and plated in 60-mm dishes 1 day prior to treatment, and then the media was replaced with media containing freshly prepared DAC (Sigma-Aldrich, St. Louis, USA) to a final concentration of 1 μM. Fresh medium containing DAC was replaced every 24 hours. After incubating with DAC for 1–7 days, the cells were harvested and counted for further assays.

### Western blot analysis

Whole-cell proteins were extracted in RIPA buffer with proteasome inhibitor. Protein content was quantified by the BCA assay (Pierce, Rockford, USA). Forty micrograms of whole cell lysates were separated by 10% SDS-PAGE and transferred to PVDF membranes. Membranes were blocked with 5% non-fat dry milk in Tris-bufferd saline with Tween 20 (TBST) for 2 hours and probed with primary antibodies diluted in TBST containing 5% milk at 4°C overnight. Blots were incubated with antibodies against human MAGE-A (6C1; Santa Cruz Biotechnology, Santa Cruz, USA), NY-ESO-1(Santa Cruz Biotechnology) and GAPDH (Abcam, Cambridge, USA). The anti-MAGE-A antibody 6C1 cross-reacts with MAGE-A1, A2, A3, A4, A6, A10 and A12. The molecular weight of MAGE-A10 is 72 kDa, and that of the rest ranges from 45–50 kDa. Membranes were washed and incubated with horseradish peroxidase-conjugated rabbit anti-mouse secondary antibody (Santa Cruz Biotechnology) for 1 hour at room temperature. Targeted proteins were visualized using an enhanced chemiluminescence (ECL) detection system (ChemiDoc™ XRS+ imaging system; BIO-RAD, Hercules, USA) and hyper-ECL film. Band analysis for gray value was performed by the Quantity One software (BIO-RAD).

### Quantitative real-time reverse transcriptase-polymerase chain reaction

Total RNA was extracted using the Trizol reagent (Takara, Shiga, Japan) and qualified by absorbance at 260nm. cDNA was synthesized from 1 μg of RNA by Takara RNA PCR kit (Takara) following the manufacturer's instructions. Real-time PCR was performed using a SYBR GREEN Master Mix (Takara) on ABI StepOnePlus System (Applied Biosystems, Warrington, UK). Primer sequences used are listed in Table 1. Samples were performed in triplicate and averaged. The relative expression level of genes was normalized to the value of GAPDH by delta-delta cycle threshold (ΔΔCT) method, allowing the calculation of differences in gene expression using the ABI software.

**Table 1:** Primer sequences used in this study

Gene	Primer Sequence	References
GAPDH	Forward: 5′-GAAGGTGAAGGTCGGAGTC-3′ Reverse: 5′-GAAGATGGTGATGGGATTTC-3′	[1]
MAGE-A1	Forward: 5′-GCTCTGTGAGGAGGCAAGG-3′ Reverse: 5′-GCAGCAGGCAGGAGTGTG-3′	[2]
MAGE-A2	Forward: 5′-ATCTGCCTGTGGGTCTTCATTG-3′ Reverse: 5′-AGCGGTCTGCTGCTCCTC-3′	[2]
MAGE-A3	Forward: 5′-TCGGTGAGGAGGCAAGGTTC-3′ Reverse: 5′-CGGGAGTGTGGGCAGGAG-3′	[2]
MAGE-A4	Forward: 5′-GAGCAGACAGGCCAACCG-3′ Reverse: 5′-AAGGACTCTGCGTCAGGC-3′	[3]
MAGE-A6	Forward: 5′-GGAAGGTGGCCAAGTTGGTTC-3′ Reverse: 5′-CCAGCTGCAAGGAATCGGAAG-3′	[4]
MAGE-A10	Forward: 5′-CACAGAGCAGCACTGAAGGAG-3′ Reverse: 5′-CTGGGTAAAGACTCACTGTCTGG-3′	[3]
MAGE-A12	Forward: 5′-CGTCGGTGGAGGGAAGCAG-3′ Reverse: 5′-GGCAGCAGGTAGGAGTGTGG-3′	[2]
NY-ESO-1	Forward: 5′-GCGGCTTCAGGGCTGAATGGATG-3′ Reverse: 5′-AAGCCGTCCTCCTCCAGCGACA-3′	[5]

All sequences were blasted (http://www.ncbi.nlm.gov/BLAST/)

REFERENCES1PattynFSpelemanFDe PaepeAVandesompeleJRTPrimerDB: the real-time PCR primer and probe databaseNucleic Acids Res2003311221231251996310.1093/nar/gkg011PMC1654582ZouCShenJTangQYangZYinJLiZXieXHuangGLevDWangJCancer-testis antigens expressed in osteosarcoma identified by gene microarray correlate with a poor patient prognosisCancer2012118184518552200916710.1002/cncr.264863De PlaenEArdenKTraversariCGaforioJJSzikoraJPDe SmetCBrasseurFvan der BruggenPLetheBLurquinCEtAStructure, chromosomal localization, and expression of 12 genes of the MAGE familyImmunogenetics199440360369792754010.1007/BF012466774Muller-RichterUDDowejkoAReutherTKleinheinzJReichertTEDriemelOAnalysis of expression profiles of MAGE-A antigens in oral squamous cell carcinoma cell linesHead Face Med20095101935871810.1186/1746-160X-5-10PMC26905795WangRFJohnstonSLZengGTopalianSLSchwartzentruberDJRosenbergSAA breast and melanoma-shared tumor antigen: T cell responses to antigenic peptides translated from different open reading framesJ Immunol1998161359836069759882

### Generation of dendritic cells and CTA specific cytotoxic T-cells

Peripheral blood mononuclear cells (PBMCs) were obtained from volunteers and isolated by Ficoll gradient centrifugation. Immaure dentritic cells (DCs) were generated from adherent cells cultured for 5 days in presence of 1000U/ml GM-CSF and 10ng/ml IL-4 (R&D Systems, Minneapolis, USA) in complete medium (RPMI 1640 supplemented with 10% FBS, 2μM glutamine and antibiotics). DCs were matured on day 5 using a cocktail consisting of 10ng/ml TNF-α, 10ng/ml IL-1β, 10ng/ml IL-6 (Gibco), and 1ug/ml PGE-2 (Sigma). From day 7, mature DCs were pulsed with multi-MAGE-A peptide (YLEYRQVPV) [18] and NY-ESO-1 peptide (SLLMWITQC) (Sangon Biotech, Shanghai, China), and subsequently used as antigen-presenting cells (APC) to stimulate CTA specific T-cells in the presence of 10ng/ml IL-7, 10ng/ml IL-15 and 50U/ml IL-2 (Gibco). The cultures were replaced weekly, and after two weeks, cells were harvested and counted.

### Flow cytometry phenotyping

*In vitro* generated DCs were assessed for surface expression using fluorochrome-conjugated monoclonal antibodies: PE conjugated anti-human CD1a, PE conjugated anti-human CD11c, FITC conjugated anti-human CD83 and APC conjugated anti-human HLA-DR (BD Biosciences, San Jose, USA). DCs were harvested and washed with cold PBS, and antibodies were added to bind at 4°C for 20 minutes in the dark. Then cells were washed twice with PBS for flow cytometry, performed with FACSCanto (BD Biosciences) flow cytometer. Data were analyzed with FlowJo software.

### IFN-γ enzyme-linked immunospot analysis

The specificity of generated CTA specific CD8+ T-cells was evaluated by IFN-γ enzyme-linked immunospot analysis (ELIspot). Cells were plated in triplicates at 50,000 cells per well of anti-IFN-γ capture antibodies coated plates. Multi-MAGE-A peptide and NY-ESO-1 peptide mixes were then added onto cells on each well, and irrelevant peptides were used as negative control. IFN-γ release was assessed, and spot forming cells were counted.

### Cytotoxic assay

MTS assay was used to evaluate the cytotoxic effects of *in vitro* generated CTA specific T-cells on osteosarcoma cells. Osteosarcoma cells were plated in triplicate into 96-well plates at 1 × 10^3^, 2 × 10^3^ and 6 × 10^3^ cells/well for 5 days, 3 days and 1 day DAC treatment respectively. Cultures containing DAC were replaced every day. Upon completion of DAC treatment, all cultures were replaced with RPMI 1640 medium supplemented with 10% FBS, and T-cells were added at 2 × 10^5^/well. After incubation for 4 hours, the supernatant was removed, and all wells were washed with PBS softly twice to remove the T-cells. The percentage of survived cells was measured using the MTS assay according to the manufacturer's instructions and quantified by determining the optical density using a microplate reader.

### Animal studies

Healthy 5-week-old female SCID mice were provided by the Experimental Animal Research Center of Zhejiang Chinese Medical University. All animal procedures were approved by the local Committee for Animal Experiments. For the purpose of *in vivo* imaging, HOS cells transfected with luciferase (HOS-Luc) and T-cells labeled with XenoLight DiR (Caliper life sciences, Hopkinton, USA) were used in the animal studies. HOS-Luc cells (5 × 10^6^ in 200μl PBS) were injected subcutaneously near the scapula of the SCID mice. To test the induction of CTAs in tumor xenografts after DAC treatment at 1μg/g of body weight [41] (dissolved in PBS and injected intraperitoneally every day for 5 days, starting at the 7^th^ day after injection of tumor cells), three mice were sacrificed after the final DAC injection. The tumor tissues were frozen with liquid nitrogen for western blotting. To examine the treatment efficacy of CTA specific T-cells in combination with DAC, we separated the mice randomly into 4 groups, each of which contained 6 mice. They were all injected with tumor cells one week before. Tumors were measured with caliper every two days. Among the 4 groups, group I were set as control, group II were mice treated with DAC and saline, group III with PBS and T-cells, and group IV with both DAC and T-cells. DAC dissolved in PBS or PBS alone was injected intraperitoneally every day. After 5 days, drug treatment was over and mice started to receive *i.v.* injection of *in vitro* generated CTA specific T-cells (50 × 10^6^ in 100μl PBS) or PBS every two days. On the 13^th^ day, *i.e.* the day after the first injection of T-cells, mice of group C and group D took isoflurane inhalation and were then imaged with an In Vivo Imaging System (IVIS Lumina Series III, Caliper life sciences). The whole treatment was finished on the 19^th^ day, and all mice were sacrificed by cervical dislocation after isoflurane inhalation. The tumor tissues were then measured and weighed. The approximate volume of tumor was calculated using the formula *V = a × b^2^/2*, where *a* is the maximal diameter of the tumor, and *b* is the minimal diameter.

### Statistical analysis

Student's *t* test was applied for statistical comparison among data of *in vitro* studies. Differences among results of *in vivo* studies were assessed using the Kruskal-Wallis H-test followed by Bonferroni's multiple comparison tests. *P* < .05 was considered significant.
